# Effects of ABO blood groups and RH-factor on COVID-19 transmission, course and outcome: A review

**DOI:** 10.3389/fmed.2022.1045060

**Published:** 2023-01-12

**Authors:** Mohammad T. Abuawwad, Mohammad J. J. Taha, Luai Abu-Ismail, Warda A. Alrubasy, Shams Khalid Sameer, Ibrahim T. Abuawwad, Yaqeen Al-Bustanji, Abdulqadir J. Nashwan

**Affiliations:** ^1^Department of Clinical Medicine, Faculty of Medicine, Cairo University, Cairo, Egypt; ^2^Department of Ophthalmology, Islamic Hospital, Amman, Jordan; ^3^Department of Clinical Medical, School of Medicine, University of Jordan, Amman, Jordan; ^4^Department of Nursing, Hamad Medical Corporation, Doha, Qatar

**Keywords:** ABO blood groups, COVID-19, hematology, RH factor, SARS-CoV-2

## Abstract

ABO and Rh blood grouping systems are two of the non-modifiable risk factors that play an important role in the susceptibility, severity and outcomes of COVID-19 infection. This review explores these associations all over the world, in an attempt to conclude a clear idea for future reference in clinical practice. In the present review, a link has been drawn between blood groups and COVID-19 transmission, course and prognosis, as literature suggests that blood group O plays a protective role against the infection, while blood group A exhibits a higher risk of exacerbation. In contrast with Rh negative individuals, Rh positive individuals are prone to more severe infection and complications, despite the fact that the underlying mechanisms of this association remain understudied. Nevertheless, the connection remains subject to controversy; since some studies report doubts about it. Thus, this association requires further investigation.

## 1. Introduction

Coronavirus disease 2019 (COVID-19), caused by the novel severe acute respiratory syndrome coronavirus 2 (SARS-CoV-2), emerged in late December 2019, causing a global pandemic as declared by World Health Organization in March 2020 (1). Since then, evidence from medical research is growing regarding the risk factors that may increase the susceptibility, morbidity and mortality of the infection which could help decrease the burden of this crisis on medical services and guide public health recommendations and interventions.

One of these risk factors is the ABO blood group system, which is considered an inheritable and a non-modifiable risk factor. The ABO system is one of the most important systems among 33 other blood group systems listed by the international society of blood transfusion, dividing blood into four groups: A, B, AB, and O (2). Blood groups are determined by carbohydrates present on the surface of red blood cells (RBCs), giving them the ability to act as receptors for different microorganisms and toxins (3). The first suggestion of a link between ABO blood group and clinical COVID-19 manifestations was reported by Zhao et al. (4). From then on, many studies tackled this association (5). Moreover, the Rhesus (RH) factor which is another RBC surface protein is suspected to have a role in the COVID-19 infection (6). As a consequence, to the aforementioned data, several hypotheses have been suggested in a trial to explain the underlying mechanisms behind this association.

This work aims at reviewing available research that investigated the correlation between ABO blood groups and RH factor with transmission, course, and outcome of COVID-19 infection. A brief worldwide overview of studies handling this topic was introduced, and the mechanisms by which this effect is exerted are described.

## 2. Method

In this research, we collected the most relevant papers to our chosen title and keywords. Search was conducted through major platforms like “Scopus”, “Google Scholar”, “Cochrane library”, and “PubMed” using the following keywords: “ABO blood group”, “blood groups”, “COVID-19”, and “SARS-CoV-2” and combinations between them. The search was conducted between 13 and 25th of August, 2022.

Our eligibility criteria are (A) Studies that investigated the relationship between COVID-19 and ABO blood groups, (B) Studies that investigated the relationship between COVID-19 and RH factors. (C) Studies published in international peer-reviewed journals and (D) English language-only. We excluded animal studies, studies writer in languages other than English, and commentary articles. Later and more robust articles were prioritized. For the section on worldwide sample, the largest and most major studies from each country were elected.

## 3. Association between blood groups and COVID-19 transmission

The relationship between ABO blood groups and SARS-CoV-2 infection susceptibility was reported at the beginning of the pandemic in China (4). It is reported that blood group A may be associated with an increased risk of infection and blood group O may play a protective role against SARS-CoV-2 transmission (4). Therefore, many studies have been conducted to investigate the relationship between ABO groups and COVID-19. A systematic review conducted by Bing-Bing Wu and his team concluded the same results; individuals with blood group A are more likely to get infected in comparison with non-A blood groups, while on the other hand, blood group O individuals are less liable to get COVID-19 (7). Several global studies support this relationship and report similar results regarding blood group A and blood group O and their impact on COVID-19 infection (4, 8, 9). In retrospect, looking back on the previous SARS-CoV-1, an association between ABO blood groups and the susceptibility to SARS-CoV-1 was also detected, as blood group O played a protective role against transmission similar to SARS-CoV-2, but the data for the effects of blood group A was insufficient as far as our search goes (10).

**Figure 1:**
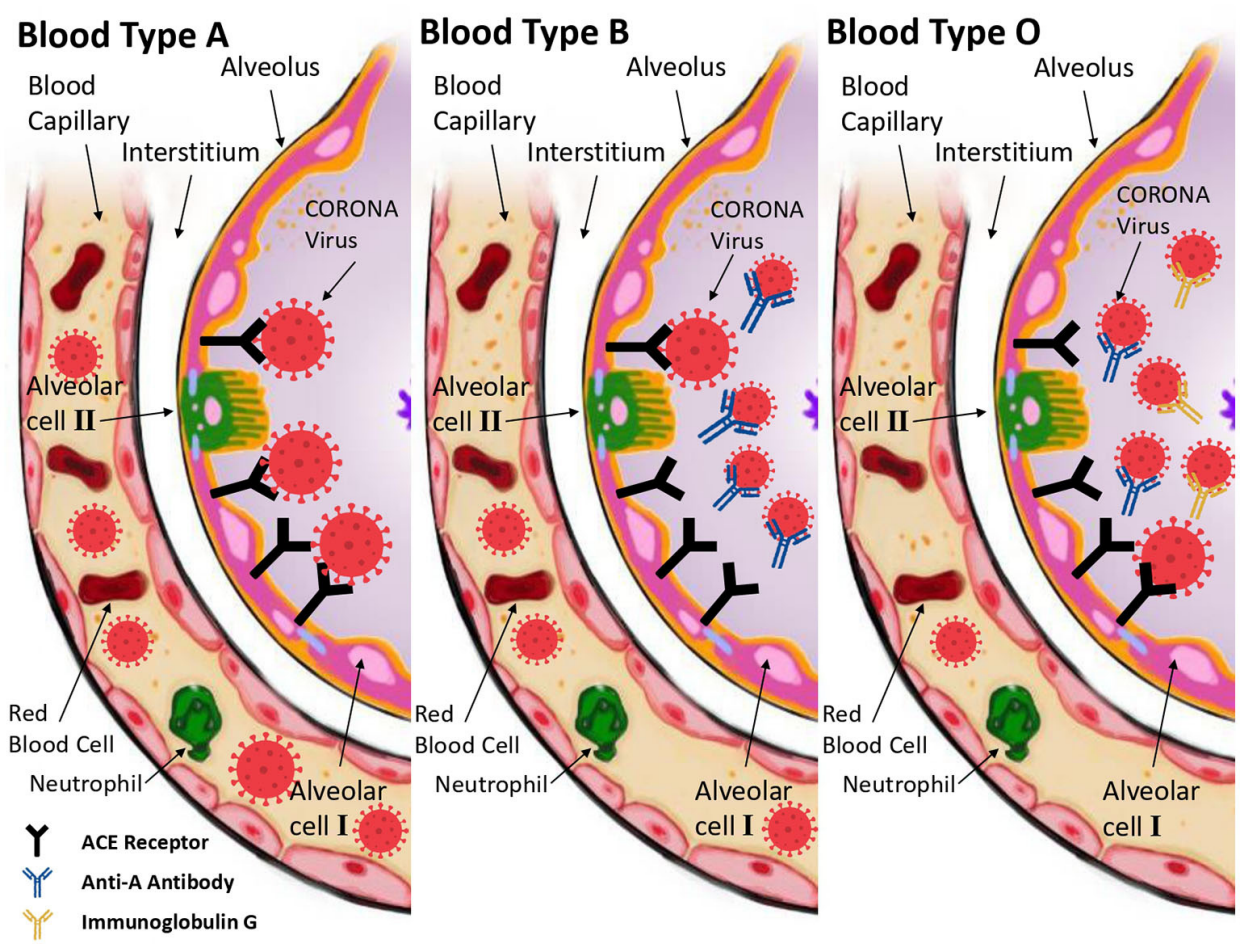
The molecular mechanism that explains how and why the ABO blood group affects a human's vulnerability to COVID-19 disease. The A blood group's lack of antibodies makes it easier for SARS-CoV-2 to enter the host cell and cause the subsequent viral infection **(Left side)**. Anti-A antibodies prevent the S protein of the virus from interacting with ACE-Receptors on the cell surface in blood group B cells **(Middle)**. Finally, blood group O provides further protection against COVID-19 infection due to the presence of anti-A antibodies and IgG antibodies **(Right side)**.

The ability of anti-A antibodies to inhibit the adhesion between SARS-CoV-2 virus with angiotensin converting enzyme 2 (ACE2)-expressing cells was one of the main hypotheses that explain this relationship (Figure 1) (11). This also sheds light on the idea that the antibody itself, not the blood group, determines the relationship. For example, both B and O blood groups have anti-A antibodies, and according to Christiane Gerard who conducted a study on the antibody itself; it was found that subjects with anti-A in serum (i.e., B and O blood groups) are significantly less reported as the COVID-19 patients than those who do not have the anti-A antibody. However, in the same study, when comparing the anti-A antibody in O and B blood groups, a significant difference was found in the anti-transmission effect where blood group O plays more protective role than blood group B, which suggests other contributing factors that relate to the increased presence of Immunoglobulin G (IgG), anti-A, and anti-B in group O plasma (Figure 1) (12). In addition, the lifestyle and the local climate besides blood type distribution may also affect the infectivity onset and initial growth rate of the virus (13).

A meta-analysis studied 10 articles and found that the susceptibility to infection by COVID-19 increases in blood group A compared to non-A types, and that this result still applies in different racial groups (5). Many other studies found the same results, but these papers reported relatively contradicting minor results regarding blood groups B and AB where they showed various effects on COVID-19 ranging from low to high risk (4, 14–16).

## 4. Effects of ABO blood groups on the course, clinical presentation, and prognosis of COVID-19

Evidence from research reported that patients with blood group A are at a higher risk of developing severe COVID-19 infection compared to the other blood groups, while patients with blood group O are at a lower risk of developing severe infection compared to the other blood groups. According to a study conducted by Heit et al., blood group O individuals have lower plasma levels of procoagulant factor VIII and Von Willebrand factor compared to other blood groups (17), rendering them less likely to develop venous thromboembolism. This is an important fact to consider when thinking about COVID-19 infection related coagulopathy and pulmonary thromboembolism, which are important issues in the context of COVID-19 infection and must be handled carefully (18). Li et al. reported in his study that individuals with blood group O are less likely to develop COVID-19 pneumonia and pulmonary microthrombi (19). On the contrary, patients with blood group A who have hypertension or other cardiovascular diseases are more likely to develop a severe form of SARS-CoV-2 infection and should receive ultra-care (20, 21).

In a study conducted in three hospitals in Wuhan, China in the period between February 1 and March 25, 2020 on patients who were diagnosed with COVID-19, resulting either in their death, or discharge from the hospital demonstrated that blood group A was associated with a higher risk of hospitalization following SARS-CoV-2 infection, while blood group O corresponded to lower risk of hospitalization (19). Another study conducted by Zhao et al. in Wuhan also found that blood group A had a higher rate of death in contrast to blood group O which was associated with a lower death rate (4).

Nevertheless, a systematic review described a significant association between COVID-19 and ABO groups, as more than a half (62.5%) of them found a better prognosis for blood group O, and almost a half (54.17%) found a worse prognosis for blood group A, yet only 33.33% reported both results together. There has also been a contradiction in the results of the other blood groups (B and AB) (22).

Overall, the respective blood group of patients are suspected to have an effect on the course of COVID-19 infection, either by prolonging/reducing their hospital stay or by imposing a more/less severe manifestations. Moreover, the outcome of the infection is also influenced by the blood type, as better prognosis was linked to blood group O, while worse outcome was associated with A blood group.

## 5. Main underlying mechanisms of association

Since the pandemic, a lot of studies have shed light on the molecular mechanisms explaining SARS-CoV-2 interaction with host cells. Viral particle entry is mediated through binding of the virus's Spike (S) protein, which is a glycoprotein emerging from the viral envelope, to the ACE2-receptors that are present on several human cells (23). Several hypotheses were suggested to explain the mechanism behind the association of ABO blood groups and SARS-CoV-2 infection. A study conducted by Cooling, proved that blood group antigens, which are determined by oligosaccharides, act as receptors for several microorganisms including coronaviruses. The study showed that blood group A cells contain an additional sugar, N-acetyl galactosamine, which is not present on blood group O cells (3). Hence, Zaidi et al. suggested that this could explain the increased cell pathogen exposure in individuals with blood group A (24).

It is reported that anti-A antibodies that are present in individuals with blood groups O and B, block the interaction between the Spike protein and ACE2 receptors, thus inhibiting the virus entry and attachment, which leads to an attenuated infectivity of the virus. Therefore, blood group O and B individuals exhibit partial or complete protection against the virus, whereas blood group A individuals have a higher risk of infection since they lack anti-A antibodies (5). Another note is that anti-A antibodies in blood group B present Immunoglobulin M (IgM) while the antibodies from group O present Immunoglobulin G (IgG), giving more protection in blood group O (12). Moreover, it was reported that non-O blood groups, specifically group A, have increased levels of angiotensin-converting enzyme (ACE), which plays a role in promotion of the inflammatory response, meaning that O blood groups have lower ACE levels and a higher protection against the virus (20).

Regarding the severity of the infection in different ABO phenotypes; blood group A and AB patients were found to have elevated levels of D-dimer, this might be of significance in the development of severe respiratory manifestations in the SARS-CoV-2 infection (25). Furthermore, blood groups O and B have reduced levels of factor VIII and von Willebrand factor. Reduced levels of these factors provide protective mechanisms against complications from SARS-CoV-2 such as pulmonary vasculopathies, therefore blood group A individuals are more susceptible to a more severe infection by SARS-CoV-2 (26). This mechanism requires further work to be better understood.

Another important aspect of association between specific ABO types and increased COVID-19 severity is the genomic aspect of each group. A few genome-wide association studies (GWAS) on the association between ABO groups and COVID-19 severity were performed. Upon studying the ABO group locus, it was found that blood group A has a higher risk of developing respiratory failure, while blood group O has a protective role (27). Similar findings were observed regarding the allelic variants of the ABO phenotype (28). A trans-ethnic GWAS of COVID-19 severity found that the allele TC is a risk allele for COVID-19, and that its carriers belong to blood groups other that O (29). On the other hand, homozygote T/T carriers who belong to blood group O are relatively protected against the severe infection.

## 6. The role of Rhesus (RH) factor

Regarding the RH factor, Rh-negative (Rh-) individuals had a lower risk of initial infection in comparison to Rh-positive (Rh+) individuals (9). This is also found in a study that reported Rh+ individuals were more likely to test positive for SARS-CoV-2 (30). Furthermore, a study on 825 hospitalized COVID-19 patients showed that 95% of patients were Rh+ (31).

RH factor also plays a role in the severity of the COVID-19 infection. Ray et al. reported that Rh– blood group is associated with lower risk of developing severe COVID-19 infection, suggesting that Rh– blood group may have a protective effect against severe SARS-CoV-2 illness (18). Similar results were reported in a study conducted by Zietz et al., where it concluded that COVID-19 patients with negative RH were less likely to develop infection, intubation, and death (9).

## 7. A worldwide comparison between different studies regarding its final results and findings

**Figure 2:**
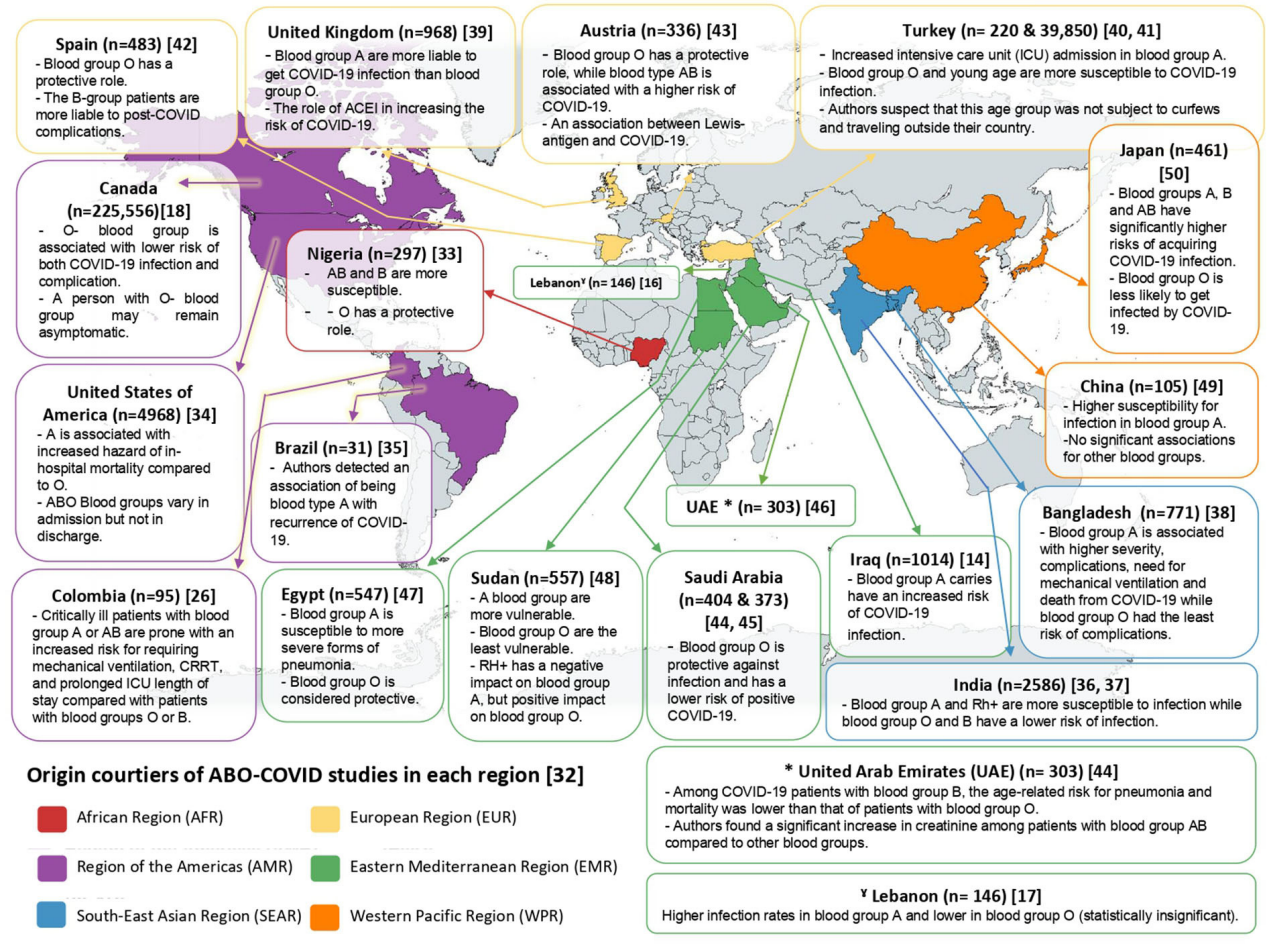
Map clarifying the distribution of studies discussing ABO-COVID relationship from each region of WHO.

Table 1 illustrates studies from different countries around the world distributed among 6 regions according to the world health organization (WHO) classification of the world (32). These studies investigated the relationship between COVID-19 and ABO blood groups among their respective populations. A map illustrating the final conclusion of each paper and its corresponding country is provided in Figure 2.

**Table 1:** A summary of population-based studies sampled from each region in the world.

**Country (32)**	**Sample Size (Positive COVID test)**	**COVID-19 cases per blood group**	**Main conclusion**
**African Region (AFR)**			
Nigeria (33)	297	A: 58 (19.1%) B: 77 (25.3%) AB: 17 (5.6%) O: 145 (47.7)	Blood groups AB and B were more susceptible to the disease. Blood group O has a protective role (Statistically insignificant finding)
**Region of the Americas (AMR)**			
United States of America (34)	4968	A: 1,473 (29.6%) B: 846 (17.0%) AB: 204 (4.1%) O: 2,445 (49.2%).	Patients vary in admission rates based on ABO groups but not in discharge. Blood type A was associated with the increased cause-specific hazard of all-cause in-hospital mortality com-pared to type O.
Canada (18)	225,556	A: 81,797 (36.3%) B: 33,536 (14.9%) AB: 10,221 (4.5%) O: 100,002 (44.3%)	O-negative blood group is associated with lower risk of both COVID-19 infection and complication. A person with O- blood group may remain asymptomatic.
Columbia (26)	95 (ICU due to COVID-19)	A: 35 (37%) B: 16 (17%) AB: 3 (3%) O: 41 (43%)	Critically ill COVID-19 patients with blood group A or AB are prone with an increased risk for requiring mechanical ventilation, CRRT, and prolonged ICU length of stay compared with patients with blood groups O or B.
Brazil (35)	31	A: 15 (48.4%) B: 4 (12.9%) AB: 1 (3.2%) O: 11 (35.5%)	Authors detected an association of being blood type A with recurrence of COVID-19.
**South-East Asian Region (SEAR)**			
India (36, 37)	2,586	A: 774 (29.93%) B: 1,081 (41.80%) AB: 183 (7.08%) O: 548 (21.19 %)	Blood group A and Rh+ are more susceptible to infection while blood group O and B have a lower risk of infection.
	509	A: 112 (22%) B: 158 (31%) AB: 183 (36%) O: 56 (11%)	Neutralizing antibodies significantly more in AB and least in O. Blood group AB are at higher risk of infection. Blood group O have lowest risk of infection.
Bangladesh (38)	771	A: 288 (37.35%) B: 134 (17.38%) AB: 204 (26.46%) O: 145 (18.81%)	Blood group A had the greatest need for supplemental oxygen and mechanical ventilation. Blood group A is associated with higher severity, complications and death from COVID-19 while blood group O had the least risk of complications.
**European Region (EUR)**			
United Kingdom (39)	968	N/A	Blood group A are more liable to get COVID-19 infection than blood group O. The role of ACEI in increasing the risk of COVID-19.
Turkey (40)	39,850	A: 15,663 (39.3%) B: 5,865 (14.7%) AB: 4,359 (10.9%) O: 13,963 (35.1%)	Increased intensive care unit (ICU) admission in blood group A.
Turkey (41)	220	A: 100 (45.45%) B:32 (14.55%) AB: 12 (5.45%) O: 76 (34.55%)	Blood group O and young age are more susceptible to COVID-19 infection. Authors suspect that this age group was not subject to curfews and traveling outside their country.
Spain (42)	483	A: 220 (45.5%) B: 44 (9.1%) AB: 19 (3.9%) O: 200 (41.4%)	Blood group O has a protective role. The B-group patients are more liable to post-COVID complications.
Austria (43)	336	A: 151 (44.9%) B: 54 (16.1%) AB: 31 (9.2%) O: 100 (29.8%)	Blood group O has a protective role, while blood type AB is associated with a higher risk of COVID-19. An association between Lewis-antigen and COVID-19.
**Eastern Mediterranean Region (EMR)**			
Saudi Arabia (44)	404	A: 103 (25.5%) B: 41 (10.1%) AB: 8 (2%) O: 252 (62.4%)	Blood group O is protective against infection and has a lower risk of positive COVID-19.
Saudi Arabia (45)	373	A: 102 (27.35%) B: 88 (23.59%) AB: 20 (5.36%) O: 163 (43.70%)	
United Arab Emirates (UAE) (46)	303	A: 84 (27.7%) B: 76 (25.1%) AB: 21 (6.9%) O: 122 (40.3%)	Among COVID-19 patients with blood group B, the age-related risk for pneumonia and mortality was lower than that of patients with blood group O. Authors found a significant increase in creatinine among patients with blood group AB compared to other blood groups.
Iraq (14)	1,014	A: 360 (35.5%) B: 221 (21.8%) AB: 109 (10.7%) O: 324 (32%)	Blood group A carries have an increased risk of COVID-19 infection.
Egypt (47)	547	A: 153 (28%) B: 139 (25.4%) AB: 28 (5.1%) O: 227 (41.5%)	Blood group A is susceptible to more severe forms of pneumonia. Blood group O is considered protective.
Lebanon (16)	146	A: 59 (40.4%) B: 25 (17.1%) AB: 10 (6.9%) O: 52 (35.6%)	Higher infection rates in blood group A and lower in blood group O (statistically insignificant).
Sudan (48)	557	A: 180 (32.3%) B: 102 (18.3) AB: 34 (6.1%) O: 241 (43.3%)	A blood group is more vulnerable to contracting the disease, whereas blood group O are the least exposed to the severe symptoms. RH+ has a negative impact on blood group A, but positive impact on blood group O.
**Western Pacific Region (WPR)**			
China (49)	105	A: 45 (42.8%) B: 28 (26.7%) AB: 9 (8.57%) O: 23 (21.9%)	Higher susceptibility for infection in blood group A. No significant associations for other blood groups.
Japan (50)	461	A: 199 (43.2%) B: 101 (21.9%) AB: 47 (10.2%) O: 114 (24.7%)	Blood groups A, B and AB have significantly higher risks of acquiring COVID-19 infection. Blood group O is less likely to get infected by COVID-19.
Total		280,580	A: 101,971 (36.3%) B: 42,668 (15.2%) AB: 15,723 (5.6%) O: 119,250 (42.5%)

According to our sample, all studies around the world supported the protective role of blood group O, except for one study from Turkey that reported opposite findings, but the authors explained that by the fact that this group of people were not subject to curfews and traveled outside their country (41). The results of the Turkish study, however, are questionable, since a major study from Canada, analyzing the data of 225,556 patients support the protective role of O blood group (18).

On the other hand, regarding A blood group, studies vary in their conclusions. Most studies support the relationship with blood group A and the increased susceptibility and severity of the COVID-19 diseases. In this context, we highlight the Turkish, as it analyzes the data of 39,850 patients and concludes that intensive care unit (ICU) admission was higher for COVID-19 patients with A blood type (40). However, a study from Nigeria reported blood groups AB and B to be more susceptible to COVID-19 rather than A (33). In addition, a study from Spain reported the role of blood group B in increasing the risk of post-COVID complications (42).

## 8. Discussion

Since the beginning of the COVID-19 pandemic, the associated risk factors that affect the transmission, course, outcome, and late complications were of interest for medical research. Both modifiable and nonmodifiable factors were extensively investigated. In this review, we try to summarize the role of one of the nonmodifiable factors. ABO blood group types have been established to have a role in many diseases, and their role in COVID-19 transmission, course, and outcome has been investigated in several previous primary and secondary research articles. In addition, we took a worldwide sample from different countries around the world according to WHO region classification, choosing the largest sample in each country of each region.

The overall outcome of this review can be summed up to the following: O blood group individuals have additional protective features against acquiring COVID-19, and against its worsening, contrary to individuals with blood group A, who are more prone to the virus's infectivity and exacerbation. Results get more confusing regarding patients with blood groups B and AB, because their associations with the incidence, course and outcome of COVID-19 are still not fully understood. Despite these findings, it is important to read these results keeping in mind the percentage of each blood group in population. A blood group has a higher frequency compared to B and AB groups, meaning that more COVID-19 cases are expected to have A blood group. The frequency of each blood group varies according to population, but A and O blood groups are generally of higher frequency, in contrast with B and AB groups, which are less prevalent (51).

Many hypotheses were proposed in literature in order to explain these results. People with blood group O were less likely to get infected with the virus, mainly due to the presence of anti-A antibodies in their serum, which block the interaction of the virus's S protein with the ACE2-receptor, thus preventing cellular entry of the virus (11). This explanation was reported in many studies, but it could not explain why blood group B does not play the same protective role as blood group O, despite the presence of anti-A antibodies in blood group B serum as well. This suggests that other factors like increased presence of IgG, anti-A and anti-B antibodies in group O plasma are involved (12). The contradiction regrading B and AB groups could be referred to the rarity of both group among population (51), therefore; a very large sample is needed to achieve a relatively robust analysis.

The RH factor, in addition to the ABO blood group, had a significant impact on COVID-19 transmission and outcome. The role of Rh+ in transmission requires further explanation and research, as systematic reviews and numerous studies have reported that Rh+ individuals are far more common than Rh– individuals (52), implying that the normal population was originally composed primarily of Rh+ individuals, making those people more vulnerable to infection. This also could be combined with the fact that there is no clear explanation why Rh– people are less likely to get infected as far as our research goes. Similar to ABO groups, the percentage of Rh+ individuals in population is significantly higher than Rh– type (51). This difference in frequency need should not be forgotten when interpreting the results of the present review.

Other studies also reported the other associated factors that may have an important role besides the blood groups. A meta-analysis conducted by Nanyang Liu found that the overall results of COVID-19 infection with blood group B in Caucasians were shown to have limited alteration (5) while in contrast Jori E. May et al. conducted a study on 165 most of them were African American and Cacusian and she found that there was no association between ABO type and admission to an intensive care unit, diagnosis of thrombosis during hospitalization, or death (53). Moreover, ABO blood groups are affected by gender too, as Muñiz-Diaz reported that the male gender is associated with worse prognosis, especially when associated with other comorbidities (21). Khalil et al. reported that male gender is not considered as a significant risk factor for developing a severe form of the disease, but had a higher incidence of infection (16). In contrast, Fan et al. reported that the association between blood group A and increased susceptibility to COVID-19 infection reaches statistical significance only in females (49).

## 9. Conclusion

We conclude that there is a strong association between ABO blood groups and COVID-19 transmission, course and outcome. Blood group O plays a protective role while blood group A is associated with an increased risk of COVID-19 infection and worse prognosis in comparison to other blood groups. To add, there are many other associated factors that may play a significant role alongside ABO blood groups, such as the RH factor. However, the role of the RH factor needs further research and investigation as its role is not yet fully understood, hence, it is highly recommended that the formerly mentioned association be considered in medical care units, and that cases are dealt with accordingly. Moreover, case-control studies targeting this issue are needed, and the lack of such studies is a limitation for understanding and addressing this topic. We suggest the following manner of benefiting from the ABO grouping when dealing with COVID-19 patients: people with blood group A should receive higher consideration if confirmed as COVID-19 positive patients, whereas those with blood group O should not hesitate to follow the protective measures against the infection, all whilst maintaining control over the cases. Individuals are also recommended to adopt a healthy lifestyle with a good nutritious diet in order to benefit maximally from the protective antibodies against the virus.

## Author contributions

MA and MT: figures design. MA, MT, WA, SS, IA, AN, and YA-B: literature search and manuscript preparation. All authors have read and agreed to the published version of the manuscript.
